# COVID-19 and Obesity: Role of Ectopic Visceral and Epicardial Adipose Tissues in Myocardial Injury

**DOI:** 10.3389/fendo.2021.726967

**Published:** 2021-08-16

**Authors:** Adèle Lasbleiz, Bénédicte Gaborit, Astrid Soghomonian, Axel Bartoli, Patricia Ancel, Alexis Jacquier, Anne Dutour

**Affiliations:** ^1^Department of Endocrinology, Metabolic Diseases and Nutrition, Pôle ENDO, APHM, Marseille, France; ^2^Aix Marseille Univ, INSERM, INRAE, C2VN, Marseille, France; ^3^Aix Marseille Univ, CNRS, CRMBM, Marseille, France; ^4^Department of Medical Imaging, Hôpital Universitaire Timone APHM, Marseille, France

**Keywords:** epicardial adipose tissue, COVID-19, obesity, cardiac injury, adipose tissue, ectopic fat, inflammation, immunity

## Abstract

In March 2020, the WHO declared coronavirus disease 2019 (COVID-19), caused by severe acute respiratory syndrome coronavirus 2 (SARS-CoV-2), a global pandemic. Obesity was soon identified as a risk factor for poor prognosis, with an increased risk of intensive care admissions and mechanical ventilation, but also of adverse cardiovascular events. Obesity is associated with adipose tissue, chronic low-grade inflammation, and immune dysregulation with hypertrophy and hyperplasia of adipocytes and overexpression of pro-inflammatory cytokines. However, to implement appropriate therapeutic strategies, exact mechanisms must be clarified. The role of white visceral adipose tissue, increased in individuals with obesity, seems important, as a viral reservoir for SARS-CoV-2 *via* angiotensin-converting enzyme 2 (ACE2) receptors. After infection of host cells, the activation of pro-inflammatory cytokines creates a setting conducive to the “cytokine storm” and macrophage activation syndrome associated with progression to acute respiratory distress syndrome. In obesity, systemic viral spread, entry, and prolonged viral shedding in already inflamed adipose tissue may spur immune responses and subsequent amplification of a cytokine cascade, causing worse outcomes. More precisely, visceral adipose tissue, more than subcutaneous fat, could predict intensive care admission; and lower density of epicardial adipose tissue (EAT) could be associated with worse outcome. EAT, an ectopic adipose tissue that surrounds the myocardium, could fuel COVID-19-induced cardiac injury and myocarditis, and extensive pneumopathy, by strong expression of inflammatory mediators that could diffuse paracrinally through the vascular wall. The purpose of this review is to ascertain what mechanisms may be involved in unfavorable prognosis among COVID-19 patients with obesity, especially cardiovascular events, emphasizing the harmful role of excess ectopic adipose tissue, particularly EAT.

## Introduction

Since December 2019, a global pandemic of coronavirus disease 2019 (COVID-19), caused by severe acute respiratory syndrome coronavirus 2 (SARS-CoV-2), first reported in Wuhan, China, has been raging (1). Obesity, whose prevalence is rising worldwide, is currently a major public health issue. It was soon recognized as a risk factor for worse outcomes of COVID-19 (2), including the occurrence of acute respiratory distress syndrome (ARDS), but also adverse cardiovascular events in up to 28% of hospitalized patients (3). The role of ectopic fat depots, especially increased amounts of epicardial adipose tissue (EAT), has drawn interest in the COVID-19 setting because this cardiac adiposity could fuel critical illness in patients with obesity. The purpose of this review is to ascertain what mechanisms may be involved in the unfavorable prognosis of COVID-19 patients with obesity, especially cardiovascular events, emphasizing the harmful role of excess ectopic adipose tissue, particularly EAT.

## COVID-19 Pathogenesis—Key Points

The mechanisms of SARS-CoV-2 viral transmission and pathogenesis are now better understood and may explain why some patients appear to be at greater risk of severe forms. SARS-CoV-2 infects the host cells by binding of the viral spike (S) proteins, present on the viral envelope, to cellular angiotensin-converting enzyme 2 (ACE2) receptors and then by employing cellular serine protease TMPRSS2 for S protein priming and plasma membrane fusion (4). This enables endocytosis of the virion and entry of the viral genome into the host cell cytoplasm, followed by endosomal acidification, viral replication, and shedding of virion particles (5). Type II alveolar cells, kidney cells, myocardial cells, nasal, ileum, esophagus epithelial cells, pancreatic cells, and, interestingly, adipocytes (6–8) have been identified with high ACE2 expression and could increase SARS-CoV-2 infection and replication as demonstrated in a mouse model and HeLa cells (9, 10). Infection results in cell apoptosis, which triggers the activation of pro-inflammatory cytokines and chemokines. It has been demonstrated that SARS-CoV-2-infected patients, especially those requiring admission to intensive care units (ICUs), have large amounts of pro-inflammatory cytokines than healthy patients without SARS-CoV-2 infection (11). One of the mechanisms explaining rapid disease progression could be the “cytokine storm”, a dysregulated, excessive systemic cytokine release (12). Studies have shown that serum levels of IL-6, tumor necrosis factor (TNF-α), granulocyte colony-stimulating factor (G-CSF), interferon-γ-inducible protein 10 (IP-10), monocyte chemoattractant protein 1 (MCP-1), or macrophage inflammatory protein 1-α, among others, are higher in patients with severe conditions (i.e., requiring transfer to an ICU or mechanical ventilation or who died) than in other infected patients (13, 14). Obesity is known to be associated with a state of chronic low-grade inflammation that might be a risk factor for developing a cytokine storm form during COVID-19 disease.

## Obesity: A Risk Factor for Bad COVID-19 Outcomes

Obesity is increasing worldwide and is today clearly recognized as a critical risk factor for various infections, post-infection complications, and mortality from severe infection (15, 16). In particular, since the 2009 influenza A H1N1 outbreak, patients with obesity have been found to be at greater risk of severe disease and have needed more mechanical ventilation (17, 18). During the COVID-19 pandemic, poor prognostic factors have emerged such as male sex, older age, diabetes mellitus, hypertension, and the presence of prior cardiovascular or respiratory disease. These factors were associated with a greater risk of developing critical or fatal conditions (2, 19). Obesity was also soon recognized as an independent risk factor associated with worse outcomes (20, 21). The United Kingdom was the first to reveal in March 2020, through a report from the Intensive Care National Audit and Research Centre (ICNARC), that two-thirds of patients who developed serious or fatal complications following infection were overweight or obese. A US study including 5,700 patients hospitalized in New York City for COVID-19 reported that the prevalence of obesity in recovered patients was twice that in the population around the hospital (41.7% *vs.* 22%) (22). A pooled meta-analysis including 19 studies showed that individuals with obesity were 113% more at risk of hospitalization (*p* < 0.0001) (23). This was confirmed by another study including 45,650 participants from nine countries worldwide and showing an odds ratio of 2.36 (95%CI: 1.37, 4.07, *P* = 0.002) for hospitalization, and 2.63 (95%CI: 1.32, 5.25, *P* = 0.006) for invasive mechanical ventilation support (24). It has also been shown that individuals with obesity are more likely to be managed in ICUs with a need for orotracheal intubation for mechanical ventilation especially if patients are young (23, 25–27). In CORONADO, a multicentric French study of COVID-19 infection in hospitalized patients with diabetes, body mass index (BMI) was the only pre-admission criterion associated with orotracheal intubation and death at D7 especially in patients younger than 75 years (28, 29). In a French cohort of 5,795 patients hospitalized for COVID-19 infection, obesity doubled mortality in all age groups (30).

## Ectopic Fat and Adipose Tissue Dysfunction: Key Elements in the Complications of Obesity

Regional distribution of adipose tissue and the development of ectopic fat are major determinants of metabolic and cardiovascular diseases (31, 32). Dysfunction of subcutaneous adipose tissue (SAT) limits its expandability and leads to ectopic fat deposition.

### Adipose Tissue Dysfunction

During weight gain, adipose tissue undergoes multiple structural and cellular remodeling processes (33) leading to a dysfunctional tissue. Firstly, during chronic positive energy balance, mature adipocytes expand, becoming hypertrophic to store more fat. If this extra energy is not used, cell numbers increase in adipose tissue, which then becomes hyperplastic (34). Hyperplastic and hypertrophic adipocytes are often hypoxic, partly explaining the development of inflammation (35). Secondly, hypoxia also induces the production of HIF-1α, which in turn leads to a potent profibrotic transcriptional program with extracellular matrix (ECM) component accumulation, leading to fibrosis and adipose tissue dysfunction (36, 37). Concurrently, immune cells infiltrate the adipose tissue, and pro-inflammatory cytokines are overexpressed (33). Under lean conditions, high M2/M1 ratio, eosinophils, and regulatory T cells, which secrete IL-4/IL-13 and IL-10, lead to an anti-inflammatory phenotype. In obesity, activation of several stress pathways such as endoplasmic reticulum stress, oxidative stress, and inflammasome (38), but also hypoxia, induces a shift in innate immunity and lymphoid cells and a modification of macrophagic signature with a rapid shift in polarization toward an M1 phenotype, associated with adipose tissue inflammation and insulin resistance (35, 39, 40). A chronic low-grade inflammation state is therefore mainly explained by immune cell imbalance in dysfunctional adipose tissue. Stressed adipocytes release free fatty acids (FFAs) and secrete chemokines that lead to inflammatory immune cell infiltration secreting pro-inflammatory cytokines (41). Intestinal microbiota dysbiosis can also trigger inflammation by activation of immune-signaling pathways (42). The dysfunction of SAT leads to the release of FFAs to peripheral organs and ectopic fat deposition such as EAT.

### Epicardial Adipose Tissue and Cardiovascular Risk

In the last decade, it has been demonstrated that ectopic fat depots localized around the heart contribute to the pathogenesis of cardiovascular disease, independently of other visceral depots (43, 44). EAT is an ectopic fat depot located between the myocardium and the visceral pericardium in close contact with coronary vessels (45). With no fascia separating the tissues, local interaction and cellular crosstalk between myocytes and adipocytes can occur. EAT is an extremely active endocrine organ with a high capacity for releasing and taking up FFAs. It is thought that EAT has protective functions as a mechanical shock absorber against pulse waves, a regulator of FFA homeostasis, and, in a more recent work, a thermogenic factor (46–49). It is a major source of adipokines, chemokines, and cytokines, interacting paracrinally or vasocrinally with vascular cells or myocytes (44). Expression and secretion of pro-inflammatory cytokines (IL-6, IL-1β, MCP-1, TNF-α, etc.) have been found to be higher in EAT than in subcutaneous fat (50), partly by the upregulation of nuclear factor κB (NF-κB) and c-Jun N-terminal kinase (JNK). It was hypothesized to accentuate vascular inflammation, plaque instability *via* apoptosis (TNF-α), and neovascularization (MCP-1).

Using a pangenomic and unbiased lipidomic approach, we previously reported that EAT has a specific transcriptomic and lipidomic signature particularly enriched in inflammation, extracellular matrix remodeling, immune signaling, thrombosis, beiging, coagulation, apoptosis, and lipotoxic pathways with an enrichment in ceramides, diglycerides, and monoglycerides compared with SAT, especially in patients with coronary artery disease (CAD) (47, 51). Furthermore, we previously demonstrated that human EAT secretome induced marked fibrosis of myocardial atria through the secretion of adipo-fibrokines, such as activin A (52). Activin A was shown to be enhanced in patients with heart failure and reduced ejection fraction and was abundantly expressed in EAT of type 2 diabetes (T2D) patients with obesity (53).

EAT thickness, volume, and density can be assessed by various imaging techniques such as echocardiography (54), computed tomography (CT), and magnetic resonance imaging (55). Higher EAT volume and lower density were associated with coronary calcification and serum levels of plaque inflammatory markers (56). EAT has been shown to be associated with CAD and the occurrence of major adverse cardiovascular events in many studies (57–60). It is correlated with the extent and severity of CAD, chest pain, unstable angina, and coronary flow reserve (61, 62) and could be a marker of the atherosclerotic burden even in asymptomatic patients (63, 64). EAT may also play a role in the development of atrial fibrillation (AF) (65) by infiltration of adipocytes in the atrial myocardium, mechanical effect on left atrial pressure stretch and wall stress, fibrosis, and inflammation, which can lead to structural and electrical remodeling and cardiac automatic system activation (44).

Obesity thus leads to an increase in ectopic fat deposition, particularly at the epicardial level, which may partly explain the increase in adverse cardiovascular events in this condition. Moreover, the pro-inflammatory phenotype of adipose tissue makes this organ a target for further immune amplification by external pathogens, such as SARS-CoV-2. In the current context of COVID-19 infection, we will see how dysfunction of the adipose tissue leads to a higher risk of severe-form COVID-19.

## Dysfunctional Adipose Tissue in Obesity: A Key to Understanding Bad Outcomes During the COVID-19 Pandemic

### Immune and Metabolic Derangement as a Possible Link to Worse Outcomes in Obesity

It has been demonstrated that host cell entry of SARS-CoV-2 depending on ACE2 receptors and overexpression of human ACE2 can increase viral infection and replication. Some studies have demonstrated that the expression of ACE2 in adipocytes is higher than that in the lungs, which can act as an important viral reservoir (7, 66). Experimental studies on mice showed an increased expression of ACE2 in adipocytes in case of a high-fat diet (67). In obesity, excess adipose tissue may thus increase SARS-CoV-2 infection and accessibility to the tissue, leading to an increased viral systemic spread, entry, and prolonged viral shedding (68), as seen during the influenza A epidemic. After infection of host cells, the recruitment of pro-inflammatory cytokines and impaired lymphocyte T cells culminates in a cytokine storm associated with progression to ARDS and multi-organ failure (13). In severe respiratory forms, patients with COVID-19 infection showed macrophage activation syndrome. There is a depletion of lymphocytes CD4 and CD8 (69) but a higher ratio of pro-inflammatory Th17 cells and high secretion of pro-inflammatory cytokines IL-2, IL-6, and TNF-α (70, 71). In obesity, dysfunctional hypertrophic adipocytes over-produce pro-inflammatory cytokines, leading to a chronic low-grade inflammation state. This in turn causes metabolic and immune derangement, making a cytokine storm more likely (72). The dysfunction of an adaptative immune system with increased pro-inflammatory LTCD4^+^ and impaired T-cell function could also increase this risk. In this regard, the PD-1/PDL-1 immune checkpoint could increase within the visceral adipose tissue (VAT) of individuals with obesity. PD-1 is expressed by T cells and interacts with receptor PDL-1 to inhibit cytotoxic T cell responses. A recent study showed that T cells of individuals with obesity increased PD-1 expression, leading to T-cell exhaustion and dysfunction (73). During severe COVID-19, the number of TCD4^+^ and TCD8^+^ is also reduced, and expression of PD-1 is increased (74). Interestingly, Alzaid et al. observed particularly low levels of cytotoxic CD8^+^ lymphocytes and increased monocyte size and monocytopenia restricted to classical CD14^Hi^ CD16^−^ monocytes, which were specifically associated with severe COVID-19 in patients with T2D requiring intensive care (75). Monocyte loss was accompanied by morphological alteration and a hyper-inflammatory expression profile consistent with the type 1 interferon pathway (IL-6, IL-8, CCL2, and INFB-1). This particular immunophenotype could be a clue to a better understanding of the increased risk of severe forms in individuals with obesity by the escape of SARS-CoV-2 from lysis.

More recently, a significant increase in IL-1β level in plasma was reported in COVID-19 patients (11), suggesting that the NOD-like receptor family pyrin domain-containing 3 (NLRP3) inflammasome might be involved in the pathogenesis of infection and lung injury. NLRP3 is a multiprotein complex present in macrophages, dendritic cells, and other non-immune cells. The activation of NLRP3 as a pivotal component of the innate immune system plays a critical role in the host defense but is also associated with metabolic and inflammatory conditions (76). During SARS-CoV-2 infection, the intense and rapid stimulation of immune system response could trigger activation of the NLRP3 inflammasome pathway and the release of its products including IL-6 and IL-1β (77), which could be involved in maintaining inflammation. Viral infection could potentiate this underlying systemic inflammatory state, which could partly explain worse outcomes in obese patients (78).

It has also been demonstrated that individuals with obesity display white adipose tissue depot in large airway walls, proportionally to BMI, which could lead to airway thickening, immune cell infiltration, and then tissue damage and fibrosis in the lungs (79, 80). Also found in the lungs, lipofibroblasts, adipose-like cells composed of lipid droplets and located in the alveolar interstitium, could transdifferentiate to myofibroblasts and lead to pulmonary fibrosis (5, 7).

There would then be a higher expression of ACE2 and TMPRSS2 in lung epithelial cells from individuals with obesity than in those without, as demonstrated *in vitro* (81).

These conditions could be another basis for the elevated occurrence of ARDS in obese individuals with obesity.

These different elements partly explain the role of adipose visceral tissue in critical COVID-19 infection, as a viral reservoir and by increasing immune responses with consequences for cytokine cascade amplification and severe forms of the disease. VAT and EAT could be markers of severity, and recent studies also show that it could be implicated in myocardial injury.

### Visceral Adipose Tissue and Epicardial Adipose Tissue as Markers of Myocardial Injury

Cardiac complications have been reported in 28% of patients hospitalized for COVID-19 infection (3, 82, 83). Myocardial injury and myocarditis with elevated troponin occurs in 7%–17% of hospitalized patients and are associated with an increased risk of adverse outcomes (84, 85). Acute myocarditis represents a significant diagnostic challenge because of its varied clinical presentations and risk of worse outcomes such as heart failure. Changes in electrocardiograms, elevated cardiac biomarkers, and impaired cardiac function should be considered as alerts pointing to acute myocarditis (86). Remarkably, no culprit injury was found in 40% of patients with COVID-19 presenting ST-elevation myocardial infarction (87), which could be promoted by hypercoagulability, endothelial dysfunction, microvascular damage, hypoxia-induced injury, myocarditis, or systemic inflammatory cytokine storm syndrome. In several studies, cardiac troponin I level was found to be associated with more severe disease and mortality, making myocardial damage a prognostic factor (88, 89). Furthermore, dysrhythmias linked to hypoxia, inflammatory stress, and therapeutics affect up to 17% of hospitalized patients (90, 91). Finally, some studies report that heart failure may be present in 23% of patients hospitalized for COVID-19, half of whom had no history of hypertension or cardiovascular disease.

The mechanisms of these cardiac events are not fully clarified, and ectopic fat and EAT could be important triggers of their development. More than just BMI, several reports have shown that VAT volume measured by CT is associated with critical illness in patients with COVID-19 entailing hospitalization (92), intensive care need, or death (93–96). According to Favre et al., a visceral fat area ≥128.5 cm^2^ was the best predictive value for severe COVID-19 (93). Further, EAT, known to be strongly correlated with VAT, has been associated with the occurrence of cardiac events in COVID-19 infection.

CT imaging of the EAT allows adipose tissue inflammation to be characterized by quantifying CT threshold attenuation. The group of Iacobellis showed that density of EAT, reflecting inflammatory changes, significantly increased with increasing COVID-19 severity compared to discharged patients (97). Furthermore, EAT mean attenuation was negatively correlated to high-sensitivity troponin T levels and peripheral oxygen saturation (97). Another international multicenter study on 109 patients showed that volume and attenuation of EAT measured by CT was associated with extent of pneumonia and were independent predictors of clinical deterioration or death (98). This study used a fully automated three-dimensional measurement of EAT and demonstrated that EAT volume can predict clinical deterioration or death independently of clinical factors such as age, diabetes, hypertension, or smoking history (99). This suggests the importance of automated measurement of EAT for COVID-19 risk stratification. An increased EAT volume was associated with lung dysfunction even in healthy individuals (100), and the close proximity of EAT to the pulmonary circulation could enable direct diffusion of inflammatory mediators. According to Wei et al., EAT volume appeared to be an independent predictor of myocardial injury in patients with COVID-19 (OR = 3.06) with a maximal cutoff value of 137.1 cm^2^ (89) after adjustment for age, weight, history of cardiovascular disease, and dyslipidemia. This work performed in a large cohort of 400 patients from six Chinese hospitals clearly indicates that EAT volume enlargement may predict the development of myocardial injury. However, the cutoff needs to be evaluated in ethnically diverse cohorts. Furthermore, EAT was significantly higher in severe cases of COVID-19 groups, i.e., with signs of respiratory distress (101). In a recent study, Iacobellis et al. showed that on 427 infected patients, use of dexamethasone reduced EAT attenuation (102). EAT could therefore also serve as a therapeutic target for anti-inflammatory treatment. All these studies indicate that EAT volume and inflammation itself are associated with COVID-19 severity and adverse cardiac events.

The mechanisms of these cardiac events are not fully elucidated, and EAT could be a clue to understanding them. First, epicardial fat cells seemed to express higher levels of ACE2 than subcutaneous fat cells, which could make them a viral reservoir in COVID-19 infection. A study on EAT and SAT biopsies from 43 patients who underwent open-heart surgery identified higher levels of ACE2 (*p* < 0.05) but lower ADAM-17 (*p* < 0.001), with its cleavage enzyme in EAT compared with subcutaneous fat. Obesity and T2D exacerbated this difference in patients with cardiovascular disease (103). In an animal study, ACE2 was upregulated in murine EAT in association with high-fat diet. Loss of ACE2 in knock-out diet-induced-obesity (ACE2KO-DIO) mice increased macrophage polarization to a pro-inflammatory phenotype and EAT inflammation compared with wild-type and control diet mice. The same study showed that in human EAT from obese patients with heart failure, ACE2 was increased and was also associated with pro-inflammatory macrophage phenotype compared with lean patients (104, 105). Voluminous and hypervascularized EAT in individuals with obesity could facilitate viral spread, immune response, and greater pro-inflammatory cytokine secretion. Volume of EAT was positively correlated with inflammatory biomarkers during COVID-19 infection in a study of 100 patients (106), with a significant positive mild association with neutrophil-to-lymphocyte ratio (*r* = 0.33, *p* = 0.001) and platelet-to-lymphocyte ratio (*r* = 0.25, *p* = 0.01) but a negative correlation with lymphocyte-to-C-reactive protein (CRP) ratio (*r* = −0.25, *p* = 0.02). Pro-inflammatory cytokines such as TNF-α and IL-6 are expressed at higher levels in EAT of individuals with obesity linked to a reduction of inotropic effect and cardiac function resulting in hypoxia and systemic myocardial inflammatory response (43). By taking advantage of more ACE2-binding sites, which ultimately lead to an augmented inflammatory signaling cascade, EAT inflammation could contribute to myocardial complications, such as myocarditis or cardiomyocyte dysfunction (107), and then heart failure. Furthermore, it has recently been shown that EAT adipocytes can release exosomes that can enter cardiac cells *via* endocytosis (105). This suggests numerous mechanisms by which EAT could impair cardiac function, particularly *via* the transfer of microRNAs from EAT to the myocardium and could help mediate SARS-CoV-2 entry into the heart, causing direct cardiac effects.

COVID-19 thus induces an immune-mediated inflammatory response, and EAT may transduce this inflammation to the heart. It can be implicated in COVID-19 myocarditis by its contiguity with the myocardium and its pro-inflammatory secretome reaching the myo-pericardium directly by the vasa vasorum and paracrinally (108–110).

EAT thus contributes to bad outcomes during COVID-19 infection. We and others have shown that EAT significantly responds to drugs targeting the fat (44). EAT not only is a marker of inflammation, but it can be a target to anti-inflammatory treatment. Further studies on the impact of COVID treatment on EAT volume and inflammation are needed.

**Figure 1:**
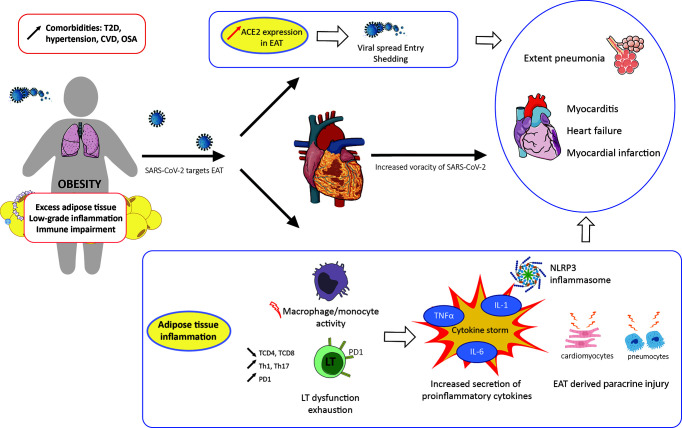
Impact of obesity and inflammation of epicardial adipose tissue on COVID-19 outcome. CVD, cardiovascular disease; EAT, epicardial adipose tissue; OSA, obstructive sleep apnea; T2D, type 2 diabetes.

All these elements are summarized in **Figure 1**.

## Conclusion

Obesity is a major risk factor for COVID-19. Identifying patients with obesity who are at high risk of ICU need is crucial. Multiple studies have demonstrated that ectopic fat accumulation, especially EAT, is a major driver of COVID-19 severity in such patients. This unique potentially inflamed EAT depot may play a direct role in COVID-19 cardiac injury, acting as a fuel through its specific anatomical contact with the myocardium and its inflammatory status. Large studies with systematic evaluation of EAT volume and CT scan attenuation together with evaluation of pulmonary involvement are needed. Deep learning algorithms leading to new fully automated three-dimensional methods for the measurement of EAT will help improve clinical risk stratification.

## Author Contributions

Conceptualization, AD and BG. Writing—original draft preparation, AL, AB, PA, AS, AD, and BG. Writing—review and editing, AJ, AD, and BG. Supervision, AD and BG. All authors contributed to the article and approved the submitted version.

## Funding

GIRCI (Groupement interrégional de recherche clinique et d'innovation) Méditerranée VALO-DATA 2020.

## Conflict of Interest

The authors declare that the research was conducted in the absence of any commercial or financial relationships that could be construed as a potential conflict of interest.

## Publisher’s Note

All claims expressed in this article are solely those of the authors and do not necessarily represent those of their affiliated organizations, or those of the publisher, the editors and the reviewers. Any product that may be evaluated in this article, or claim that may be made by its manufacturer, is not guaranteed or endorsed by the publisher.
